# The Effect of Music Therapy on Perceived Pain, Mental Health, Vital Signs, and Medication Usage of Burn Patients Hospitalized in the Intensive Care Unit: A Randomized Controlled Feasibility Study Protocol

**DOI:** 10.3389/fpsyt.2021.714209

**Published:** 2021-10-18

**Authors:** Mark Ettenberger, Rafael Maya, Andrés Salgado-Vasco, Sofia Monsalve-Duarte, William Betancourt-Zapata, Nicolas Suarez-Cañon, Sergio Prieto-Garces, Juliana Marín-Sánchez, Viviana Gómez-Ortega, Mario Valderrama

**Affiliations:** ^1^Department of Music Therapy, University Hospital Fundación Santa Fe de Bogotá, Bogotá, Colombia; ^2^Department of Biomedical Engineering, University Los Andes, Bogotá, Colombia; ^3^Department of Critical Care, University Hospital Fundación Santa Fe de Bogotá, Bogotá, Colombia; ^4^Burn Unit, University Hospital Fundación Santa Fe de Bogotá, Bogotá, Colombia

**Keywords:** music therapy, pain, burn patient, intensive care unit, mental health, medication usage

## Abstract

**Background:** Burn patients experience major physiological and psychological stressors during treatment and rehabilitation, including elevated levels of pain, anxiety, stress, or depression. Music interventions inclusive of music therapy (MT) have been shown to improve such symptoms, but rigorous clinical trials investigating specific music therapy methods in adult burn patients are scarce.

**Methods:** This is a single center Randomized Controlled Trial (RCT) protocol with two parallel arms. Participants are 81 adult burn patients admitted to the Intensive Care Unit (ICU) of the University Hospital Fundación Santa Fe de Bogotá in Colombia. The intervention consists of a Music Assisted Relaxation (MAR) protocol, a music therapy technique composed of entrained live music combined with a guided relaxation and/or the use of imagery. The effects of the MAR will be compared to a control group (treatment as usual) over a period of maximum 2 weeks or six interventions. The primary outcome measure is perceived background pain, as measured with a Visual Analog Scale (VAS) before and after each intervention. Secondary outcomes are anxiety and depression levels; vital signs; and the use of pain medication. Additionally, some patients in the intervention group will be invited to participate in electroencephalography, electromyography, and electrocardiography recordings during the MAR.

**Discussion:** This study protocol follows the SPIRIT guidelines for defining items of clinical trials and is the first study in Colombia to evaluate the effects of music therapy for adult burn patients. With this RCT it is hoped to gather new knowledge about the potential of music therapy to help critical care patients cope and recover from their injuries during the hospitalization in the ICU.

**Trial registration:** www.clinicaltrials.gov, Identifier: NCT04571255.

**Protocol version:** V1.0, May 24th 2021

## Introduction

Burn wounds are among the most painful and traumatic injuries that a person can experience and provide multiple challenges for both patients and health care professionals (1). Effective fluid resuscitation, preventing burn shock, and recovering cardiovascular and metabolic balance are some of the initial priorities for burn patients in the Intensive Care Unit (ICU) (2, 3). Early wound healing and the restoration of skin tissue are further medical procedures on which the success for survival and recovery depend on (4). Pain is a symptom that accompanies burn patients during all phases of treatment and rehabilitation and successful pain management is key to both physical and mental health (5). In the ICU, the evolution and trajectory of pain in burn patients is difficult to predict, and frequently the pain is intense and persistent (6, 7). Pain in burn patients can be categorized according to four types (8):

- First, the background pain resulting from the injuries and the destruction of skin tissue. Many times, this pain is of moderate or low intensity, but usually remains over time.
- Second, many patients experience intense procedural pain during medical and therapeutic procedures, such as wound debridement, dressing change, or during physical therapy, among others.
- Third, breakthrough pain, which can occur at any time.
- And fourth, the post-operative pain that is associated with wounds caused by skin extraction and grafting.

It is widely recognized that pain perception is based on an individual experience that affects the patient's physiological, mental, emotional, and social aspects of life (9). The International Association for the Study of Pain (IASP) defines pain as “an unpleasant sensory or emotional experience associated with actual or potential tissue damage, or described in terms of such damage” and concludes that "pain is always subjective” (10). Consequently, biopsychosocial models in treating pain in burn patients have been put forward (11, 12).

Additionally, there are also psychological challenges that burn patients must face, such as elevated levels of anxiety, stress, depression, insomnia, and changes in body identity (13). This is important, since pain perception is mediated by psychological distress in the short- and long term (14–17). One study found that 68.5% of burn patients still experience moderate pain and 42.1% live with high levels of anxiety and depression 1 year after hospital discharge (18). An adequate pain management is therefore paramount for burn patients and leads to a better recovery and overall quality of life (19). Pharmacological treatment is usually the main pillar of pain management in burn patients, but complementary therapies are widely used and recommended (20, 21). Music therapy (provided by a credentialed music therapist) and music medicine (provided by other health care professionals) interventions have been used in critical care for many years, and positive physiological and psychological effects have been reported across an array of populations (22–26). Pain is a frequently used outcome measure in different medical settings and recent reviews confirm the effectiveness of music interventions in reducing pain levels (27–29). For burn patients, a former meta-analysis showed positive effects on pain, anxiety, and heart rate (30) and a more recent systematic review and meta-analysis found statistically significant effects for pain, anxiety, and relaxation levels (31). However, most studies with adult burn patients focus on recorded music listening protocols for procedural pain, indicating a lack of RCTs investigating live music approaches for other types of pain. Non-procedural pain in adult burn patients was investigated only in two previous studies, both of which applied recorded music via an MP3 player for 20 min, once a day, for three consecutive days (32, 33). Three RCTs were found that used live music approaches, but two of them were conducted with pediatric patients (34, 35) and one with both pediatric and adult participants (36). In general, receptive music interventions such as music guided/assisted relaxation or distraction techniques, are much more common in burn patients compared to active music therapy experiences.

This study protocol aims at filling some of the current gaps in knowledge by measuring the effects of a live Music Assisted Relaxation (MAR) protocol—provided by a certified music therapist—on background pain, mental health, vital signs, and the use of pain medication in adult burn patients.

## Objectives

The primary objective of this study is to evaluate the effect of a MAR protocol compared to treatment as usual on perceived background pain in adult burn patients admitted to the Intensive Care Unit of the University Hospital Fundación Santa Fe de Bogotá.

Secondary objectives are:

- To examine the effects of the MAR on the patients' anxiety and depression levels.
- To examine the effects of the MAR on the patients' vital signs (heart rate, respiratory rate, oxygen saturation, blood pressure).
- To examine the effects of the MAR on the patients' medication usage (complementary or rescue doses).
- To examine medical and social factors, which might be associated with the effects of the MAR.
- To describe the effects of the MAR on neurophysiological parameters in a few patients in the intervention group (exploratory part of the study).

We hypothesize that the MAR protocol plus treatment as usual—as compared to treatment as usual alone—will lead to lower levels of perceived background pain and will positively impact the patients' mental health, vital signs, and medication usage.

## Methods and Analysis

### Study Design and Setting

**Figure 1:**
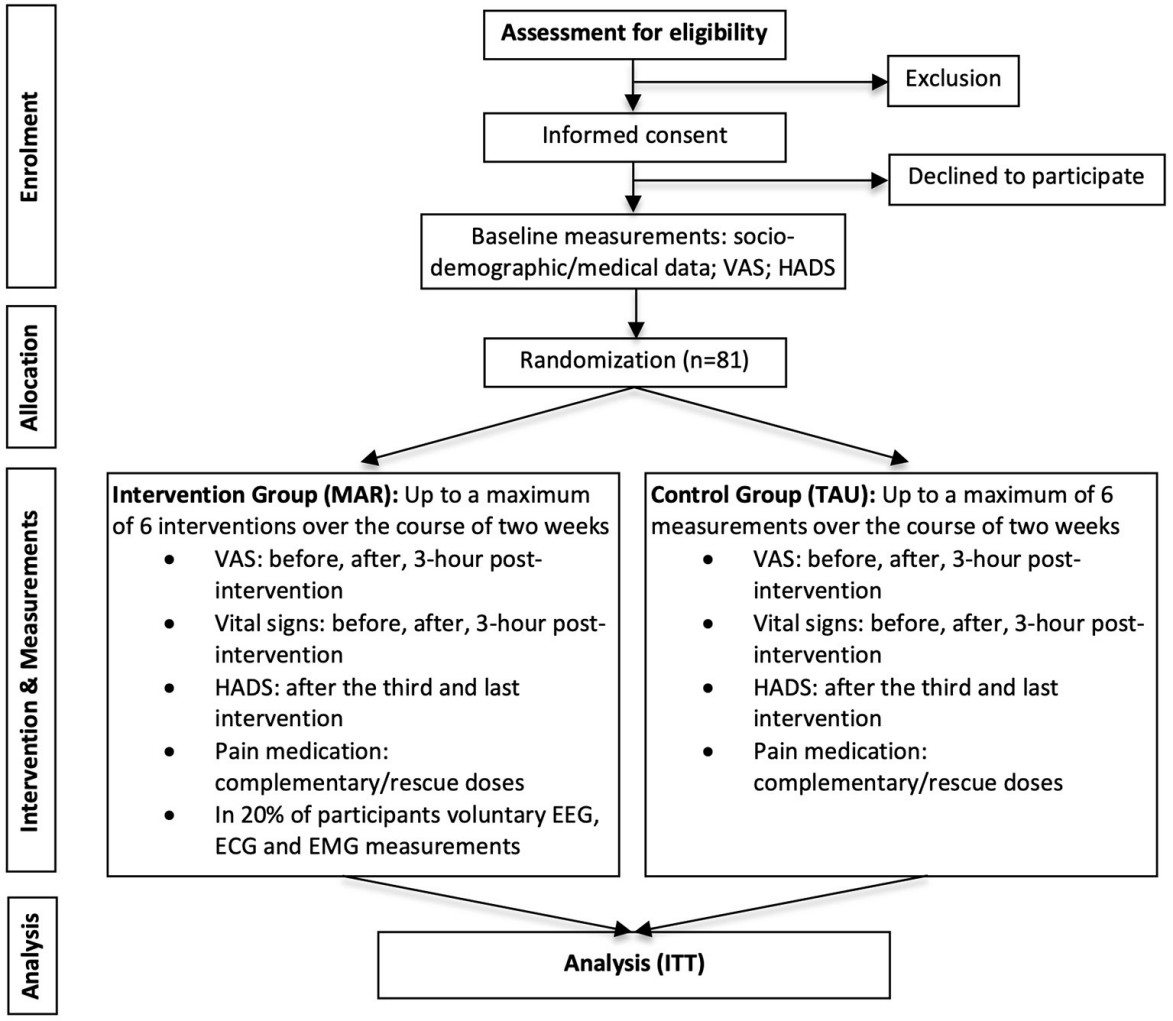
Flow-diagram. Flow-diagram adapted from Schulz et al. (34). VAS, Visual Analog Scale; MAR, Music Assisted Relaxation; TAU, Treatment as usual; EEG, Electroencephalography; ECG, Electrocardiography; EMG, Electromyography; ITT, Intention-to-treat.

This study protocol follows the SPIRIT guidelines for defining standard protocol items for clinical trials (37). This study is a single center Randomized Controlled Trial (RCT) with two parallel arms (38) and takes place at the Adults Intensive Care Unit of the University Hospital Fundación Santa Fe de Bogotá (FSFB), Colombia. Figure 1 shows the flow-diagram of the study (39).

### Inclusion Criteria and Exclusion Criteria

Inclusion criteria are burn patients of legal adult age admitted to the ICU of the University Hospital Fundación Santa Fe de Bogotá; and an estimated hospitalization of >7 days. Exclusion criteria are patients with known psychiatric disorders; patients with cognitive disabilities; and sedated and/or mechanically ventilated patients.

### Participants and Sample Size Calculation

The sample size was calculated according to the Asymptotic normal method (40). Obtaining a power of 80% and taking a *p*-value of 5%, the number of participants expected to have in the study is *n* = 81, which is similar to other studies in the field (30, 31). The formula used for calculating the sample size (40, 41) is:


$$\begin{array}{l} n=\frac{{\left[Z\left(\alpha /2\right)\sqrt{\left(2P\left(1-P\right)\right)}+Z\left(\beta \right)\sqrt{\left(P1\left(1-P1\right)+P2\left(1-P2\right)\right)}\right]}^{2}}{{\left(P1-P2\right)}^{2}} \end{array}$$

Where *P* = 0.5^*^(P1 + P2), Power = 1-β and Z(α/2) and Z(β) are taken from the z table. For this particular case, P1 corresponds to the expected outcome in the control group and P2 the expected outcome in the intervention group. The primary outcome measure is the Visual Analog Scale (VAS) that consists of 10 points (100%) and when considering a clinically significant decrease and difference of 2 points (20%), these values would correspond to 80 and 60%, respectively (P1: 80 and P2: 60). Replacing these values in the above equation, the sample size is:


$$\begin{array}{l} n=\frac{{\left[1.96\sqrt{\left((2.07)*(1.07)\right)}+0.842\sqrt{\left(0.8*(1-0.8)+0.6*(1-0.6)\right)}\right]}^{2}}{{\left(0.2\right)}^{2}}\\ \;=81.25\;\approx \;81 \end{array}$$

### Randomization and Group Allocation

Randomization takes place in the form of assigning the participants according to a set of randomized numbers generated by a computer software (Excel 2020, Microsoft Cooperation). The randomization ratio is 1:1. Once randomization is executed, sealed envelopes are used for each participant and group allocation is performed accordingly.

### Blinding

Due to the nature of the intervention, blinding participants to the result of the randomization is not feasible. However, data collection takes place by blinded research assistants and data analysis will also take place by researchers blinded to the outcomes of the randomization.

### Intervention

Participants in the intervention group will take part in a Music Assisted Relaxation (MAR) protocol. MAR is a music therapy technique that includes listening to live music based on the principles of entrainment (42, 43), combined with deep diaphragmatic breathing and guided relaxation and imagery. In the first step, the procedure of the intervention is explained to the patient, and he/she is asked to close the eyes or focus on a fixed point on the ceiling or wall. This is followed by a verbal introduction focusing on body and respiratory awareness. A mental image is then introduced, for example: sitting on a beach watching the waves of the sea; being on top of a mountain contemplating the horizon; imagining a personalized safe and comfortable place; among others. The patient is asked to breathe with the music while concentrating on the imagery and sounds. While the improvised music can change according to each patient and situation, it usually consists of a slow tempo, simple harmonic structures, free-flowing melodies, avoiding large intervals or abrupt changes in tonalities. The musical instruments used are a classical guitar with nylon strings (Yamaha C-40), an ocean drum (a drum with a double skin over which small metal pallets role imitating the sound of waves) and a Samafon (an instrument consisting of aluminum/copper tubes of different sizes that are stuck with a soft mallet producing long-lasting single or combined tones). Once the music stops, the patient is asked to become aware and the experience during the session is verbally elaborated. Each intervention will last between 30 and 40 min and will be delivered three times per week from the day of study entry up to a maximum of 2 weeks or six interventions in total. The time frame and frequency of the interventions has been chosen to stay close to daily clinical practice, in which the music therapy team of the FSFB usually sees patients 2-3 times per week. This has been a therapy frequency working well in terms of being able to offer a process-oriented approach, but not to overstrain patients due to their numerous commitments with other therapies and medical procedures. With respect to the time frame, burn patients stay in the ICU of the FSFB an average of 13,9 days, with 42% staying up to 1 week, 19% up to 2 weeks, and 38% more than 2 weeks. However, depending on the medical condition, it might take a couple of days or more from admission onwards until the patient is medically stable enough to start therapy. Thus, an intervention period of 2 weeks was chosen to capture most of the population. The musical instruments are disinfected and cleaned according to a protocol that has been published elsewhere (44).

Participants in the control group receive treatment as usual (TAU) according to the attending physician. An environmental control will be imposed for the control group to equalize basic conditions during data collection including promoting a resting posture, interrupting care procedures unless absolutely necessary, and keeping the room doors closed.

### Data Collection and Outcome Measures

Basic socio-demographic and medical data will be collected from all participants, including age; gender; educational level; marital status; level of support by the patient's social system; medical diagnosis; type and location of the burns; and depth and surface % of the burns.

### Primary Outcome Measure

Primary outcome measure is a Visual Analog Scale (VAS) of 0-10. 0 indicates a state without any pain and 10 indicates the most severe pain possible. Each number from 0 to 10 is distributed in a line 1 cm apart and the patient can mark the intensity of pain felt at the time of filling out the VAS. The VAS will be applied at baseline, and every time before, after, and at 3 h post-each intervention. Period of data collection: maximum 2 weeks from the point of study entry.

### Secondary Outcome Measures

- The Colombian version of the Hospital Anxiety and Depression Scale (HADS) (45) will be applied to measure anxiety and depression levels of the participants. The HADS consists of a 7-item anxiety subscale and a 7-item depression subscale rated on a four-point Likert scale. The higher the total points on each scale, the greater the risk of a depressive or anxiety disorder, with a maximum score of 21 for each subscale. The HADS scale will be applied at baseline, after the third, and after the last intervention. Period of data collection: maximum 2 weeks from the point of study entry.
- Vital signs (heart rate, respiratory rate, oxygen saturation, blood pressure) will be measured via hospital monitors before the intervention, after the intervention, and at 3 h post-intervention. Period of data collection: maximum 2 weeks from the point of study entry.
- The use of pain medication refers to the frequency and dose of additional analgesic medications requested by the patient during the study period (complementary or rescue doses). The total amount of dosage and frequency will be calculated by summing up individual records. Period of data collection: maximum 2 weeks from the point of study entry.

### Other Outcome Measures

In about 20% of the patients in the intervention group (approx. eight participants), additional Electroencephalogram (EEG), Electrocardiogram (ECG) and Electromyography (EMG) recordings will be made. Since this is an exploratory feature of the study, participation in the EEG, ECG, and EMG measurements is voluntary and depends on the location of the burn wounds, the clinical stability of the patient, and is defined together with the attending physician. The sample size of 20% was chosen based on an estimation of potential participants without any head/face/neck burn injuries, in which the application of electrodes would not be feasible. As the aim of the MAR is to help patients achieve relaxation, the hypothesis for these measurements is that this will lead to (1) an increase in Alpha and Theta brain waves, both of which correspond to deep relaxation and inward-focused states of mind; (2) a reduction in muscular tone; and (3) changes in the high frequency (HF) and low frequency (LF) components and in the LF/HF ratio indicating a parasympathetic activation. All these physiological variables will be acquired simultaneously with a Micromed LTM64 (Micromed S.P.A., Italy) acquisition system, with a sampling frequency of 512 Hz. The Micromed LTM64 is of clinical quality and has the due approval of the Colombian National Institute for Drug and Food Safety (20090486-2015). For the EEG recordings, the international 10-20 scalp system setup will be used, but the final number of electrodes will depend on medical approval for each patient. From these signals, we will assess power changes in the physiological Theta (4-8 Hz), Alpha (8-12 Hz) and Beta (12-30 Hz) frequency bands, associated with brain relaxation and engagement, respectively (46, 47). The ECG will be acquired using the lead II bipolar montage with two electrodes located bilaterally in the upper chest or both arms depending on the possibilities or limitations of each patient and on his/her medical condition. From the ECG we will measure changes in heart rate and Heart Rate Variability (HRV) associated with the intervention. For this last, we will assess contributions from the sympathetic and parasympathetic branches of the autonomic nervous system through the LF and HF components from the spectral analysis of the tacogram (48). The EMG will be recorded to evaluate changes in muscular tone associated with the intervention, using a bipolar montage with two electrodes located in the chin (when possible). Muscular tone activations will be assessed through changes in amplitude of the EMG signal.

### Participant Timeline

The schedule of enrolment, interventions and assessments is shown in the following Table 1:

**Table 1:** Schedule for enrolment, interventions, and assessments.

	**Study period**						
	**Enrolment**	**Allocation**	**Post-allocation**				**Closeout**
**Timepoints**	**–t_**1**_**	**t_**0**_**	**t_**1**_**	**t_**2**_**	**t_**3**_**	**etc**.	**t_**x**_**
**Enrolment**							
Eligibility screen	**x**						
Informed consent	**x**						
Allocation		**x**					
**Interventions**							
Intervention group			**x**	**x**	**x**	**x**	
Control group			**x**	**x**	**x**	**x**	
Assessments							
Baseline measures:	**x**						
Socio-demographic/Medical data	**x**						
VAS	**x**						
HADS							
**Outcome measures**							
VAS			**x**	**x**	**x**	**x**	**x**
HADS					**x**		**x**
Vital signs			**x**	**x**	**x**	**x**	**x**
Medication usage			**x**	**x**	**x**	**x**	**x**

### Data Management and Statistical Analysis

Data collection and storing will be done manually and later transcribed to an electronic database. The accuracy of data transcription will be checked by randomly selecting 20% of the participants for whom a double-data entry procedure will be implemented as a means for control of potential errors while transcribing the results from the paper questionnaires to the electronic database. The original questionnaires will be stored in a locked shelf inside the ICU and digital data storing and processing follows the institutional guidelines for data security, including a password protected laptop, encrypted files, and using secure methods of file transfers.

Data analysis will be performed by analysts blinded to the randomization outcomes and based on an intention-to-treat analysis (ITT) (49). Basic socio-demographic and medical data will be analyzed using descriptive statistics and compared between the two groups to detect any differences in the population. For the differences of the main outcome measure (pain) and the secondary outcome measures (anxiety and depression, vital signs, use of analgesics), ANOVA (analysis of variance), the *p*-value test, and the Mann-Whitney *U*-test will be performed. When analyzing the data, a univariate analysis will be carried out in which the frequency (absolute and relative), percentages and confidence intervals of the different qualitative variables will be established. Subsequently, the association between the different variables will be evaluated through the Chi-square χ^2^ test. Additionally, the Shapiro Wilk test will be used to determine the distribution of the quantitative variables. Once this result is obtained, we will seek to determine the mean, standard deviation, central tendency, and dispersion for the variables of normal distribution, while the median and interquartile range will be sought for those of non-normal distribution. These variables will be evaluated using the Student's *t*-test or the Mann-Whitney *U*-test according to the distribution. The results will be considered statistically significant when observing a value of *p* < 0.05 (5%). Finally, a multivariate analysis will be carried out where logistic regression will be used to determine the contribution of each of the different quantitative variables in terms of the observed results.

### Ethics and Dissemination

The study was approved by the hospital's ethics committee (CCEI-11234-2019/CCEI-11971-2020) and is registered in Clinicaltrials.gov (NCT04571255). All participants will sign an informed consent to participate in the study, including two additional witnesses. Dissemination of the study results will be realized locally, nationally and internationally through publications, posters sessions and oral presentations at conferences.

## Discussion

This feasibility study investigates the effects of a live MAR protocol provided by a certified music therapist on background pain, anxiety and depression, vital signs, and medication intake in adult burn patients. Other outcome measures correspond to the exploratory feature of this study including EEG, EMG and ECG measurements in 20% of the participants in the intervention group. Pain and impaired mental health are frequent symptoms in burn patients and can negatively affect clinical trajectory and recovery during hospitalization in the ICU and beyond. Music therapy is a non-invasive and cost-effective therapy that has been shown to reduce pain and anxiety levels in several clinical populations, including burn patients (30, 31). MAR techniques are widely used by both music therapists and other health care professionals and have been proven to be effective for a variety of conditions, such as sleep difficulties in adults and elders (50), reducing arousal due to stress (51) or treating depression (52). In pediatric burn patients, a MAR protocol was successfully implemented in a previous RCT (53). As mentioned in the introduction section, MAR and other receptive music listening techniques are much more common compared to interactive music making in this population, which was applied in only two previous RCTs (34, 36). This is because burns of the hand occur more often than burns in other areas of the body (54), which limits the holding and manipulating of musical instruments. But also in case hands and arms are not directly affected by the wounds, movements and engaging in physical activity often causes pain for burn patients. Thus, music listening protocols are clearly indicated in this population and have been chosen also for this study.

The current MAR protocol extends previously used interventions by explicitly using live music based on the principles of entrainment. Entrainment—the conscious synchronization and modification of musical elements in relation to biological rhythms of the patient—can further help to individualize the music and has been shown to improve well-being and reduce symptoms in palliative care (55), pediatric surgery (56) or patients with chronic pain (57) for example. A recent study on dynamic rhythmic entrainment found an increase in peripheral blood flow and greater subjective well-being in healthy individuals for entrained music vs. fixed-beat music (58). To our knowledge, this is the first RCT in adult burn patients using a live MAR protocol specifically focusing on entrainment. From other studies it is known that neuronal network oscillations can be entrained by external auditory stimuli and may enhance cognitive and motor function or sleep quality for example (59). Thus, one of the aims of the exploratory part of this study including EEG, EMG, and ECG measurements, is to further explain and/or uncover the basic mechanisms of live and entrained music as a potential stimulus that may help adult burn patients regulate their pain perception. In fact, pain perception has been correlated with changes in brain activation at different cortical regions and is associated with specific physiological frequency bands. For instance, several studies have noted that long lasting pain or tonic pain can induce a reduction in the Alpha frequency band, which can be correlated with the intensity of pain (60). Nevertheless, as the Alpha activity can also be decreased by several other mental tasks, its specificity to pain related processing is still unclear. In the few studies that have addressed correlations between chronic pain and associated brain oscillations, an abnormal decrease of Theta rhythms together with an increase in Alpha and Beta activities in central and frontal regions of the brain have been reported (61). These changes might reflect an alteration of the thalamocortical loops related to the generation of these rhythms, which also play an important role in the integration of sensory information (62). Similarly, they can manifest a modification in the flow of information among cortical networks implicated in pain processing (63). In the case of music, several studies have reported that exposure to music stimuli can entrain brain oscillations in different ranges (64). Moreover, some studies have shown that pleasant music can have a positive effect in external attentional networks and a reduction in internal mind-wandering processes (65). Consequently, we hypothesize that the MAR intervention applied in this study could modulate the expression of particular frequency rhythms having an important role in reducing the network activity implicated in pain perception.

While it is known that music itself can release neurotransmitters and hormones related to pain and stress relief (66), the trauma that many burn patients experience and the inherent link between pain and mental health requires the presence of credentialed professionals, such as music therapists. Profound relaxation can help in self-regulation but may sometimes evoke undesired emotions or thoughts. Stressing the importance of the therapeutic relationship applies also for this study, and time for verbal processing after the MAR will be used to elaborate the experiences during the intervention. While the length of the ICU stay for burn patients can fluctuate, we tried to offer a process-oriented protocol with a maximum of six interventions over the course of 2 weeks. This may not seem a lot, but to our knowledge, all other RCTs on music or music therapy interventions used single interventions or interventions over a few consecutive days) (30, 31).

Another new feature of this study is the focus on background pain instead of procedural pain. While background pain is usually lower in intensity, it is of longer duration and may thus alter pain memory in the long-term (6). This is important, since pain chronification may increase the already higher risks of burn patients in developing mental health impairments, such as acute stress disorder (ASD), post-traumatic stress disorder (PTSD), elevated stress levels, depression, and insomnia (6, 67). Especially in times of an ongoing opioid crisis, complementary treatments to pharmacological strategies for pain management in burn patients are needed not only for procedural pain, but also for background pain (68). In a previous meta-analysis of music interventions and pain in different medical settings, Lee (28) found a statistically significant reduction in opioid intake. However, in the three RCTs found with adult burn however, this result has not been replicated (33, 69, 70). In this study protocol we will try to evaluate total opioid intake during the intervention period, specifically in relation to rescue doses requested by the patients.

## Potential Limitations

We have also identified several potential limitations of this study protocol. First, background pain is influenced by many different factors, such as the medical condition of the patient, the number of procedures, mental health, and social support, among others. This means that a diversity of factors can potentially affect the pain perception of patients. This might be different during procedural pain, in which a more direct relationship between the procedure and the pain experience exists and thus immediate effects of music interventions might be observed more consistently. However, as mentioned at the end of the previous section, background pain in burn patients is an under-investigated topic and may be highly relevant for long-term well-being and health. While an accumulative effect of the MAR intervention over time may lead to an improved relaxation response and coping mechanisms and thus to reduced pain levels and improved mental health, little is known about the ideal dosage or frequency of music interventions. Formal moderator/mediator analyses in the fields of music therapy and music medicine may help in addressing such issues and are clearly needed (71–73).

Another limitation might be the broad inclusion criteria, which could affect the statistical power of the study. This was chosen since recruitment during COVID pandemic might be slower than expected, as ICU beds usually are prioritized for COVID patients during each infection peak. Thus, to keep recruitment within a feasible time frame, this study does not focus on a specific patient group within the burn population.

And third, other outcome measures including EEG, EMG, and ECG measurements are an exploratory feature of this study and due to the small sample size, the results can thus not be generalized. However, we consider them as important first steps in materializing the need for describing potential neurophysiological pathways and mechanisms of live MAR interventions based on the principles of entrainment.

## Conclusion

This is the first RCT on music therapy and pain management for adult burn patients in Colombia—and to our knowledge—also in South America. It is hoped that the results of this study will help gain new knowledge about complementary therapies for burn patients in ICU settings and provide new insights into the effectiveness and the underlying mechanisms of music in health care contexts.

## Ethics Statement

The studies involving human participants were reviewed and approved by Ethics Committee of the University Hospital Fundación Santa Fe de Bogotá, CCEI-11234-2019/CCEI-11971-2020. The patients/participants provided their written informed consent to participate in this study.

## Author Contributions

ME, RM, AS-V, MV, and SP-G conceived the study and developed the study design. ME drafted the manuscript. MV provided expertise for conducting primary statistical analysis for EEG results. SM-D, WB-Z, NS-C, JM-S, and VG-O contributed to the study design. All authors approved the final manuscript.

## Conflict of Interest

The authors declare that the research was conducted in the absence of any commercial or financial relationships that could be construed as a potential conflict of interest.

## Publisher's Note

All claims expressed in this article are solely those of the authors and do not necessarily represent those of their affiliated organizations, or those of the publisher, the editors and the reviewers. Any product that may be evaluated in this article, or claim that may be made by its manufacturer, is not guaranteed or endorsed by the publisher.
